# Hemipteran defensive odors trigger predictable color biases in jumping spider predators

**DOI:** 10.1038/s41598-020-78952-5

**Published:** 2020-12-14

**Authors:** Michael E. Vickers, Lisa A. Taylor

**Affiliations:** 1 Entomology and Nematology Department, University of Florida, 1881 Natural Area Drive, Gainesville, FL 32611 USA; 2 Florida Museum of Natural History, University of Florida, 3215 Hull Road, Gainesville, FL 32611 USA

**Keywords:** Behavioural ecology, Ecology, Evolution

## Abstract

Multimodal warning displays often pair one signal modality (odor) with a second modality (color) to avoid predation. Experiments with bird predators suggest these signal components interact synergistically, with aversive odors triggering otherwise hidden aversions to particular prey colors. In a recent study, this phenomenon was found in a jumping spider (*Habronattus trimaculatus*), with the defensive odor from a coreid bug (*Acanthocephala femorata*) triggering an aversion to red. Here, we explore how generalizable this phenomenon is by giving *H. trimaculatus* the choice between red or black prey in the presence or absence of defensive odors secreted from (1) eastern leaf-footed bugs (*Leptoglossus phyllopus*, Hemiptera), (2) grass stinkbugs (*Mormidea pama*, Hemiptera), (3) Asian ladybird beetles (*Harmonia axyridis*, Coleoptera), and (4) eastern lubber grasshoppers (*Romalea microptera*, Orthoptera). As expected, in the presence of the hemipteran odors, spiders were less likely to attack red prey (compared to no odor). Unexpectedly, the beetle and grasshopper odors did not bias spiders away from red. Our results with the hemipteran odors were unique to red; follow-up experiments indicated that these odors did not affect biases for/against green prey. We discuss our findings in the context of generalized predator foraging behavior and the functions of multimodal warning displays.

## Introduction

Many aposematic prey use multimodal warning displays that pair bright colors with aversive odors or harsh sounds; these individual signal components presumably work together to enhance the effectiveness of the overall display and reduce predation, but how they work together is the subject of much recent interest^1^. The field of predator psychology (a field of animal behavior that aims to understand how predators make decisions about the prey they eat) has a long history of addressing such questions^2–7^. One hypothesis that has received substantial support is that these different signal components interact synergistically, with one component of the signal (typically an odor) triggering an otherwise hidden aversion to another component of the signal (such as color); this phenomenon is also called olfactory priming^1,8–15^. Interestingly, in many cases, the odor must be novel to the predator for it to have this priming effect on color; odors that have been experienced before by an individual predator often don’t have the same effect^8,10–14^. In addition, these synergistic effects of odor and color are not just limited to ecologically relevant chemicals produced by insects for defense; the same effects have been found even when using other novel odors that are not associated with insect prey (e.g., almond oil^8^; ethyl acetate and wintergreen oil^12^). Yet, not all novel odors studied have this effect (e.g., vanilla oil and thiazole^8^); why only some novel odors (and not others) cause this effect is not yet clear.

While the synergistic effects of odor and color may explain the general diversity and form of many multimodal warning displays in nature, this phenomenon has primarily been examined using insectivorous birds as predators (as in all of the literature cited above). This prompts us to ask whether other predators also respond similarly to colors and odors in prey and, ultimately, whether this can explain the evolution of multimodal animal defenses more broadly. Non-avian predators, especially terrestrial invertebrates, feed on large numbers of insects in nature^16^ but have largely been ignored in the broader discussion of predator psychology^2–7^. This is despite similar color biases, learning capacities, and responses to odors between birds and many invertebrate predators^17–23^.

Recent work with jumping spiders (family Salticidae) has shown that, in at least one case, spiders respond to odor/color combinations in similar ways to the bird examples cited above. When presented with a defensive odor from a chemically-defended coreid bug (Order Hemiptera), *Habronattus trimaculatus* spiders showed fewer attacks on the color red (compared with black); the result of this previous study was predicted a priori based on the bird literature, as red is a color that is often associated with aposematic prey^18^. When this experiment was repeated using colors not associated with aposematic prey (green and black), the odor had no effect on color preferences^18^. Additionally, as in the bird literature, this pattern was only found when the odor was novel to the spiders; after spiders were experienced with the odor, it no longer affected their response to red coloration^18^. This was the first time that this phenomenon had been observed in an arthropod predator, despite multiple studies showing this and similar phenomena in bird predators (reviewed and cited above). One question that remained after this previous study was whether the synergistic effect of color and odor is a general feature of *Habronattus* foraging and predator psychology (as it seems to be in birds), or whether this response is limited to one specific coreid odor.

Like other jumping spiders, *Habronattus* are voracious predators^25^ with exceptional visual acuity in their large forward-facing principal eyes^26^. Recent work suggests that spiders in this genus likely have the ability to distinguish a wide range of colors from UV to red^27^ and behavioral work suggests that they attend to both color and odor cues while foraging^18–20,24^. They are common in a variety of habitat types^28^ where they likely encounter potential insect prey with combinations of defensive odors and colors. These combinations are particularly striking in many taxonomic orders of insects that share the same habitats as *Habronattus*, such as the Hemiptera (true bugs^29^), Coleoptera (beetles^30^), and Orthoptera (grasshoppers, crickets, and katydids^1^). In a recent review of multimodal displays, sixty insect species across six orders were identified as having noxious odors that are combined with visual warning signals^1^. While *Habronattus* will leap aggressively onto tiny prey when attacking, they often attack larger or riskier prey more cautiously, sometimes even lunging at the prey and touching it before attacking (pers. obs.). This latter type of interaction might allow the prey insect to release a warning odor that would prime the spider to attend to color in making a subsequent decision to attack.

Here we examine how defensive odors from several species of chemically-defended insects affect prey color biases, and particularly aversions to the color red, in *Habronattus trimaculatus*. We hypothesized a priori that the odor and color would interact synergistically. Specifically, we predicted that spiders exposed to these defensive odors would show increased aversions to red-colored prey (because red is often associated with aposematic prey^31^). We also predicted that exposure to these odors would have no effect on preferences or aversions to the color green (which is not often associated with toxicity^31^). We selected four defensive odors from chemically-defended insect prey to include in our study. We first tested the odor from a coreid bug (*Leptoglossus phyllopus*, eastern leaf-footed bug, Experiment 1) because we wanted to replicate our previous study^18^ using another coreid odor (see quasireplication^32^). Upon finding nearly identical results to our expectations (see “Results”), we went on to replicate this study a second time using a more distantly related hemipteran (*Mormidea pama*, grass stinkbug, Experiment 2). We then replicated this two more times using odors from insects from two additional taxonomic orders (Coleoptera: *Harmonia axyridis*, Asian ladybird beetle, Experiment 3 and Orthoptera: *Romalea microptera*, eastern lubber grasshopper, Experiment 4). This study provides novel insights into how predator psychology in non-avian predators shapes the defenses of aposematic prey.

## Methods

### Study species

We collected *H. trimaculatus* (n = 240) from Gainesville and Geneva, FL, USA. We fed spiders approximately their own mass in juvenile crickets (*Gryllodes sigillatus*) 3 × per week and housed them in individual plastic cages following previously described methods^20^. We used spiders in experiments between 14 and 25 days after collecting them. No spider was used in more than one experiment or tested more than once within an experiment. We estimated the body length of all spiders immediately before they entered an experiment (by measuring from the tip of their carapace to the end of their abdomen, to the nearest mm). This enabled us to confirm that the spiders in different treatment groups within each experiment did not differ by size.

### Red versus black choice tests in the presence/absence of odor

**Figure 1:**
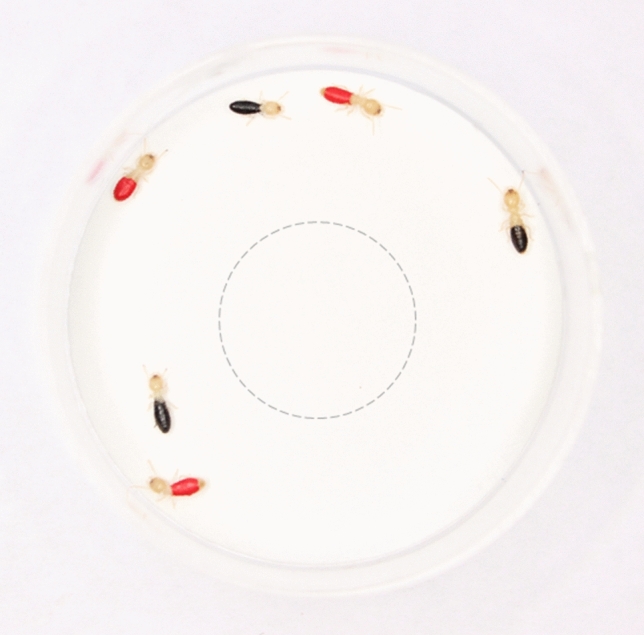
Testing arena with painted termites used in color preference tests. The arena was a round 9 cm diameter plastic petri dish lined with white filter paper. In the center of the arena, we placed a clear 3.5 cm diameter acclimation chamber (indicated by the dotted line). We released spiders from this acclimation chamber during choice tests to allow them to forage on the surrounding termites.

We replicated the methods of Vickers and Taylor^18^ across four experiments (see below). In each experiment, spiders were given choice tests with live termites (*Reticulitermes flavipes*) that had the dorsal surface of their abdomens color-manipulated with either red or black paint, applied with a toothpick (Testor Corporation, Rockford, IL, USA, red: 1150-RM11501-0611, black: 1149-RM11491-0611, Fig. 1). We used the same paint colors used in Vickers and Taylor^18^; see Fig. S1 for the spectral properties of these paints when applied to live termites. Previous studies using this color manipulation technique found no differences in movement rates between painted and unpainted termites^18^. The enamel paint does not emit any noticeable odor (to us) when dry and because all termites were painted (either red or black), any differences between groups should be driven by differences in color rather than differences in paint odor.

During prey choice tests, the spiders were randomly assigned to one of two groups. They were either (1) tested for their color preferences in the presence of a defensive odor from a chemically-defended insect (see details below) or (2) tested for their color preferences in the absence of this odor. The defensive odors used in experiments came from the following four chemically-defended insect taxa: Experiment (1) *Leptoglossus phyllopus* (eastern leaf-footed bug, Hemiptera: Coreidae), Experiment (2) *Mormidea pama* (grass stinkbug, Hemiptera: Pentatomidae), Experiment (3) *Harmonia axyridis* (Asian ladybeetle, Coleoptera: Coccinellidae), and Experiment (4) *Romalea microptera* (eastern lubber grasshopper, Orthoptera: Acrididae).

We chose these four chemically-defended taxa strategically. Although the odors from these taxa vary widely in their chemical composition (hemipterans^29^, Asian ladybeetle^33^, eastern lubber grasshopper^34^) they are all thought to be aversive to predators generally^35–40^. Their color patterns are varied, but all have some degree of conspicuous markings that seem likely to have an aposematic function (eastern leaf-footed bug: white band on dark brown body and orange on dorsal abdomen that is revealed when wings are raised; grass stinkbug: white lines and spots on black body and orange legs; Asian ladybeetle: range of spotted color forms that include both red and black; eastern lubber grasshopper: typically yellow body with black markings and red color patch on hind wings when exposed (pers. obs.); see color images in Fig. S2). Despite these differences in chemical defense and color patterns, we had no a priori reason to expect the odors from any of these insects to show stronger (or different) effects compared with others. We first tested the coreid (Experiment 1) because we wanted to replicate our previous study that found that defensive odors from another coreid caused spiders to reduce their attacks on the color red^18^. Upon finding nearly identical results to our expectations (see “Results”), we went on to replicate this study again using a more distantly related hemipteran (Experiment 2). After again finding similar patterns (see “Results”), we went on to replicate this study two more times, this time beyond the Hemiptera, using insects from two additional taxonomic orders (Experiments 3 and 4).

All four of these prey species are sympatric with *H. trimaculatus* and can even be found in the same habitats (pers. obs.). However, it is unlikely that our field-collected spiders would have any previous experience with the particular defensive odors used in this study, at least in the context of predation. This is because the adult hemipterans are much larger than the spiders (eastern leaf-footed bugs: > 16 mm, grass stinkbugs: 7.5 mm); if the spiders had any predatory experiences with these species, it would likely have only been with juveniles that produce different defensive odors than the adults^29,41^. The adult ladybird beetle is within the size range that the spiders might attack, but *H. trimaculatus* very rarely attacks beetles (pers. obs.), perhaps due to the physical protection of their hard elytra. And finally, even the smallest first instar nymphs of the lubber grasshoppers are quite large (10-12 mm in length)^42^ , reducing the likelihood of predation attempts. However, because *H. trimaculatus* will occasionally take prey up to twice their own body size (pers. obs.), we can’t completely rule out the possibility that they experienced these odors (or similar odors) in the field, but we expect these experiences to be relatively rare. Prior work suggests that jumping spiders have short-term memories of experiences with chemically-defended prey^20,43^; taken together, all of this suggests that these odors were most likely novel to the spiders when presented in experiments. Because *H. trimaculatus* is likely not a primary predator of these particular prey species, our experiments here focus on the broad question of whether insect defensive odors, generally, can shift the color biases of generalist predators.

To collect the defensive odors from the two hemipteran species, we used similar methods described in Vickers and Taylor^18^ where we placed each bug in a 3.5 cm diameter petri dish with filter paper lining the bottom and shook it for approximately 10 s. When disturbed, both the leaf-footed bugs and the stink bugs readily released their odors by spraying them from their thoracic scent glands^29,44^. For the Asian ladybird beetles, we used forceps to gently pull on their legs, which caused “reflex bleeding”^33^, whereby odorous liquid was released onto the filter paper. Finally, for the lubber grasshoppers, we picked them up by the thorax using our forefinger and thumb, holding and covering the wings, causing them to release a foamy and foul-smelling liquid from their meta-thoracic spiracles^34^; the liquid was retrieved by holding a piece of filter paper up to the spiracles. In all four cases, we could see the insects releasing copious amounts of liquid. The liquid was readily absorbed by the filter paper, and in every instance, the filter paper emitted a strong odor after collection. We did not sex the insects before collecting defensive secretions. However, even if the sexes produce different pheromones for reproduction, males and females are thought to produce the same defensive odors and these are released in copious amounts when disturbed^33,45–47^. In every instance of collecting these secretions, we observed liquid being released onto the filter paper and it emitted a strong odor after collection.

We collected all insects from the field just prior to odor collection with the exception of the lubber grasshoppers*.* Unlike the other three species which produce their defenses independent of diet, lubber grasshoppers are generalist herbivores that sequester chemical defense from their diet^36^. We know that individuals fed wild onions are particularly well defended^36^. Therefore, to standardize their chemical defense, we maintained them on a continuous diet of spring onions for approximately 11 weeks before using them for experiments.

To conduct our color choice tests, we presented spiders with three red and three black termites to choose from within a 9 cm diameter testing arena (following methods from Vickers and Taylor^18^, Fig. 1). First, spiders acclimated for 10 min in a filter paper lined central chamber (a clear 3.5 cm petri dish). For half of the spiders, the filter paper contained the defensive odor (as described above), while the other half of the spiders had filter paper without the odor. For the duration of the choice tests, the filter paper remained within the testing arena. The floor of both the acclimation chamber and the arena were covered with white filter paper which provided a consistent visual background as well as serving as the absorbent substrate for introducing the odor. The walls of the arena were clear. During the 10-min acclimation period, the spiders could view (but not contact) the termites moving around them in the arena.

After the 10-min acclimation period, spiders were released and given an additional 10 min to attack a termite. Spiders were observed directly, and we recorded (1) the orientation time (the time it took for them to orient in the direction of the first termite; jumping spiders will orient their bodies towards prey that get their attention using their forward-facing principle eyes^26^), (2) the color of the termite they first oriented to, (3) the time to took them to attack the first termite, and (4) the color of the termite attacked. We also recorded the “evaluation time” by subtracting the time to attack from the orientation time. We ended the trial once the first termite was attacked or if no attack occurred within the 10-min trial. Because termites are palatable prey for these spiders, all termites that were attacked in these tests were quickly consumed.

We ran all tests in the laboratory in an area adjacent to two large windows (1.5 × 0.9 m and 2.8 × 5.0 m). In *Habronattus* jumping spiders color vision seems to be light-limited^27,48^; therefore, we conducted choice tests between 10:00–18:00 h on sunny days when the testing arena was illuminated with natural sunlight. We tested spiders prior to being fed on their regularly scheduled feeding day resulting in a two-day period between feeding and testing. Previous work has shown that this feeding regime results in spiders that are hungry enough to attack termites during the choice tests but that are not so hungry that they will simply attack the first encountered prey item (i.e., they are sufficiently choosy)^18^. *H. trimaculatus* are generalist predators that feed on a wide variety of prey, including termites, in the field (pers. obs.).

### Green versus black choice tests in the presence/absence of odor

In our previous study^18^, we found that spiders in the presence of a defensive odor were more likely to avoid red prey, but that, as expected, odor had no effect on their responses to green prey. We wanted to replicate this study design to determine if any patterns of odor-triggered color aversions found here were unique to the color red (i.e., a common aposematic warning color), or if the same odor would trigger avoidance of a non-warning color (e.g., green). We performed the same experiments described above (Experiments 1 through 4) using new groups of field-collected spiders, giving them choices between green and black termites in the presence and/or absence of the same four defensive odors. As in Vickers and Taylor^18^, we used a green paint that was similar in the brightness to the red paint used above; we created this paint by mixing white and green paint (Testor Corporation, Rockford, IL, USA, white: 1168-RM11681-0611, green: 1124-RM11241-0611) until the green mixture did not differ in overall brightness from the red paint (t_420_ = 0.12, *p* = 0.45, Fig. S1). We wanted the red and green paint to be similar in brightness so that any differences in our experimental outcomes could be attributed to chromatic rather than achromatic (i.e., brightness) differences between the paint colors. However, because our understanding of the *Habronattus* visual system is still in its infancy^27^, we don’t yet have the visual models that would allow us to create perfectly brightness-matched red and green termites (as viewed by the *Habronattus* visual system); despite these limitations, our approach should minimize the differences in brightness as much as is currently feasible.

### Statistical analyses

For all experiments (Experiments 1–4, including both red vs. black and green vs. black tests), basic data analyses were identical^18^. To test our focal hypothesis, we performed likelihood ratio χ^2^ tests to determine if the presence or absence of the defensive odors affected the spiders’ color preferences in both our red vs. black and green vs. black choice tests. We then used likelihood χ^2^ tests to determine if there was evidence of color biases (i.e., whether attack rates on the two colors differed from 50/50) within each group. We then we used additional exploratory *post-hoc* tests to help us better interpret those results. Specifically, to determine if the presence or absence of the odor influenced the color that first got the spider’s attention, we used a likelihood ratio χ^2^ test to examine whether odor influenced the color that the spider first oriented to. Additionally, we used likelihood ratio χ^2^ tests to determine if the presence of the odor influenced (1) the orientation time (i.e., time it took spiders to orient to the termites), (2) the latency to attack the prey, or (3) the evaluation time (the time between orientation and attack).

In *H. trimaculatus* (as in many other jumping spiders), males and females look similar until they reach sexual maturity; therefore, we grouped spiders into stage/sex categories (i.e., adult female, adult male, juvenile), and tested whether this or their body size (i.e., body length) had an effect on color preferences in both red versus black and green versus black color choice tests. We had no a priori reason to expect body size or sex/stage to affect color preferences; these tests were simply exploratory. All analyses were conducted using JMP Pro (version 12.0.1).

## Results

### Experiment 1: Color choice tests in the presence/absence of odor from eastern leaf-footed bugs

#### Red versus black tests

**Figure 2:**
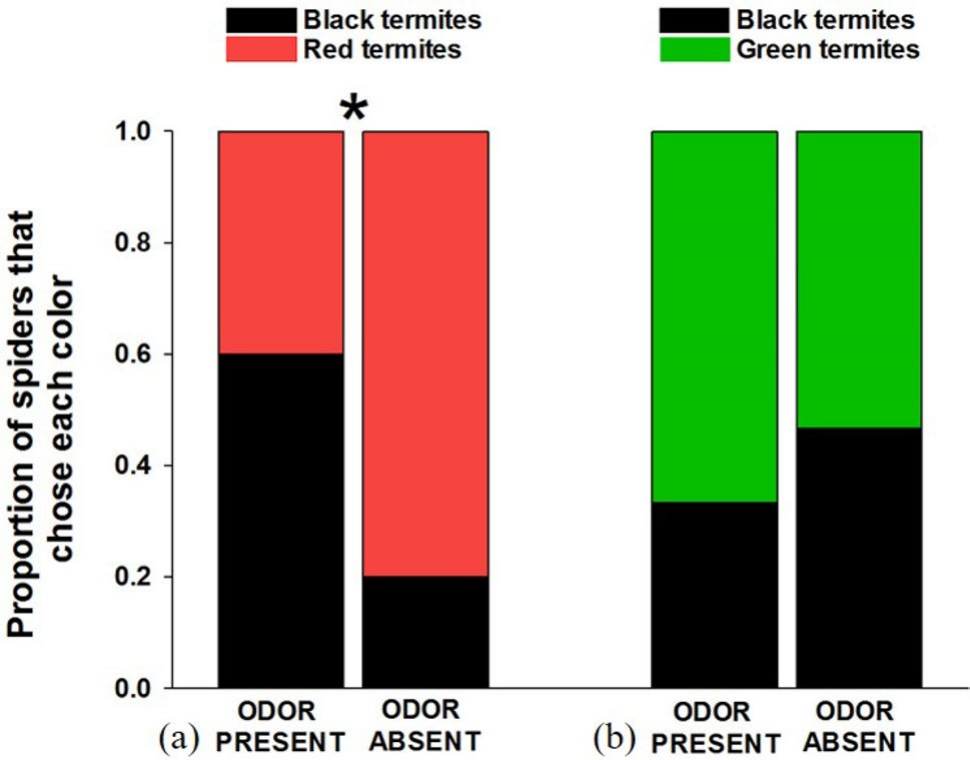
Results of choice tests between (**a**) red- and black-painted termites, and (**b**) green- and black-painted termites in the presence or absence of a defensive odor from eastern leaf-footed bugs (*Leptoglossus phyllopus)*.

As expected, we found that spiders (n = 30; 4 female, 5 male, 21 juvenile) were less likely to attack red termites when the coreid odor was present compared to when odor was absent (*X*^2^ = 5.178, *p* = 0.023; Fig. 2a). In the absence of odor, spiders were biased in their attacks toward red termites (*X*^2^ = 5.782, *p* = 0.016) but there was no evidence of a color bias when odor was present (*X*^2^ = 0.604, *p* = 0.437).

The presence of the odor did not significantly affect which color the spiders first oriented to (although there was a non-significant trend whereby spiders tended to orient towards black termites first, *X*^2^ = 3.690, *p* = 0.054). The presence of the odor had no effect on the time it took spiders to orient towards the first termite (*F*_1,29_ = 0.076, *p* = 0.784), the time it took them to attack the first termite (*F*_1,29_ = 0.038, *p* = 0.845), nor the spiders’ time to evaluate prey before attacking (*F*_1,29_ = 0.009, *p* = 0.925). Finally, we did not detect effects of sex/stage (*X*^2^ = 1.372, *p* = 0.504) or size (*X*^2^ = 0.255, *p* = 0.613) on color preferences.

#### Green versus black tests

The presence of the coreid odor did not affect the spiders’ preferences when given choices between green and black prey (n = 30; 3 female, 27 juvenile; *X*^2^ = 0.558, *p* = 0.455; Fig. 2b). There was no evidence of color biases when the odor was either absent (*X*^2^ = 0.067, *p* = 0.800) nor present (*X*^2^ = 1.700, *p* = 0.192).

The presence of the odor did not affect the color that the spider first oriented to (*X*^2^ = 0.540, *p* = 0.463), the time that it took to orient to the first termite (*F*_1,29_ = 1.311, *p* = 0.262), the time to attack the first termite (*F*_1,29_ = 0.006, *p* = 0.938), nor the spiders’ time to evaluate prey before attacking (*F*_1,29_ = 0.380, *p* = 0.543).

We found that neither sex/stage (*X*^2^ = 0.967, *p* = 0.325) nor size (*X*^2^ = 1.005, *p* = 0.316) of the spiders affected their color preferences.

### Experiment 2: Color choice tests in the presence/absence of odor from grass stinkbugs

#### Red versus black tests

**Figure 3:**
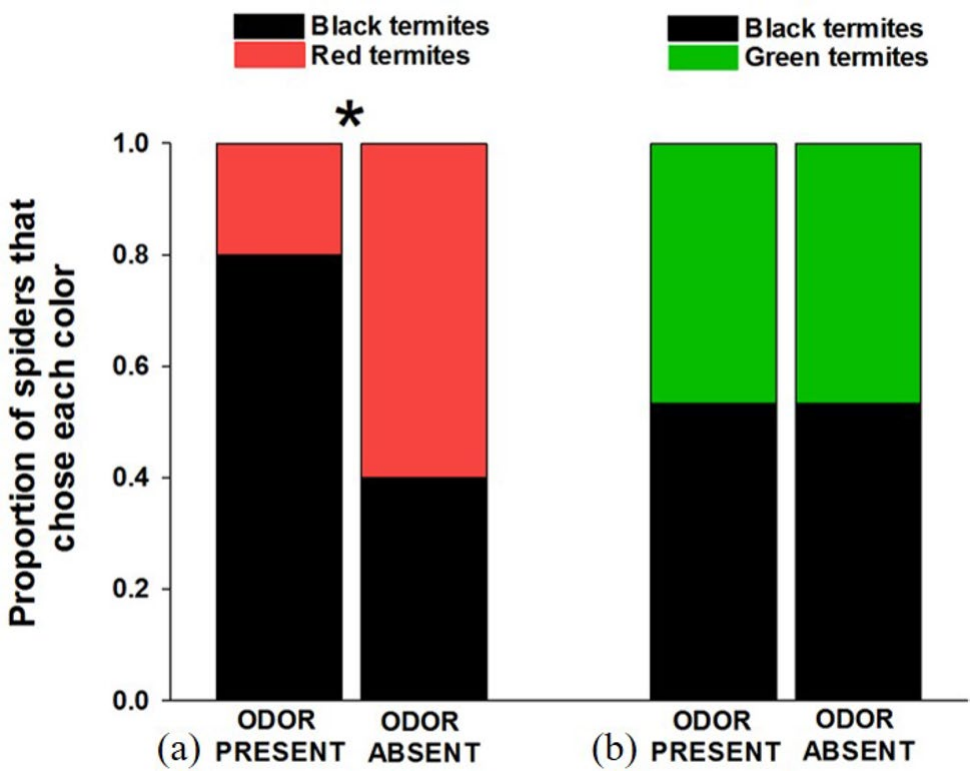
Results of choice tests between (**a**) red- and black-painted termites, and (**b**) green- and black-painted termites in the presence or absence of a defensive odor from grass stinkbugs (*Mormidea pama*).

As in Experiment 1, we again found that spiders (n = 30; 9 female, 5 male, 16 juvenile) were more likely to avoid attacking red termites when the stinkbug odor was present compared to when it was absent (*X*^2^ = 5.178, *p* = 0.023; Fig. 3a). We found a bias against red prey (*X*^2^ = 5.782, *p* = 0.016) when the odor was present, but not when it was absent (*X*^2^ = 0.604, *p* = 0.437).

When the odor was present the spiders were less likely to direct their first orientations towards red termites compared with when the odor was absent (*X*^2^ = 4.144, *p* = 0.042). The presence of the odor had no effect on the time it took spiders to orient towards a termite (*F*_1,29_ = 1.115, *p* = 0.299), attack a termite (*F*_1,29_ = 0.916, *p* = 0.346), or evaluate termites before attack (*F*_1,29_ = 0.167, *p* = 0.685). We did not detect effects of sex/stage on color preferences (*X*^2^ = 1.023, *p* = 0.600) or size of spiders on their color preferences (*X*^2^ = 0.103, *p* = 0.748).

#### Green versus black tests

Spiders in green versus black choice tests did not alter their color preferences in the presence or absence of the stinkbug odor (n = 30; 3 female, 8 male, 19 juvenile; *X*^2^ = 0.000, *p* = 0.999; Fig. 3b). There was no evidence of color biases when the odor was either absent (*X*^2^ = 0.067, *p* = 0.800) nor present (*X*^*2*^ = 0.067*, p* = 0.800).

The presence of the odor did not affect the color that the spider first oriented to (*X*^2^ = 1.231, *p* = 0.267), the time that it took to orient to the first termite (*F*_1,29_ = 0.509, *p* = 0.481), the time to attack the first termite (*F*_1,29_ = 0.173, *p* = 0.680), nor the spiders’ time to evaluate prey before attacking (*F*_1,29_ = 0.418, *p* = 0.523).

We did not detect effects of sex/stage (*X*^2^ = 4.583, *p* = 0.101) or size of spiders (*X*^2^ = 0.655, *p* = 0.316) on their color preferences.

### Experiment 3: Color choice tests in the presence/absence of odor from Asian ladybird beetles

#### Red versus black tests

**Figure 4:**
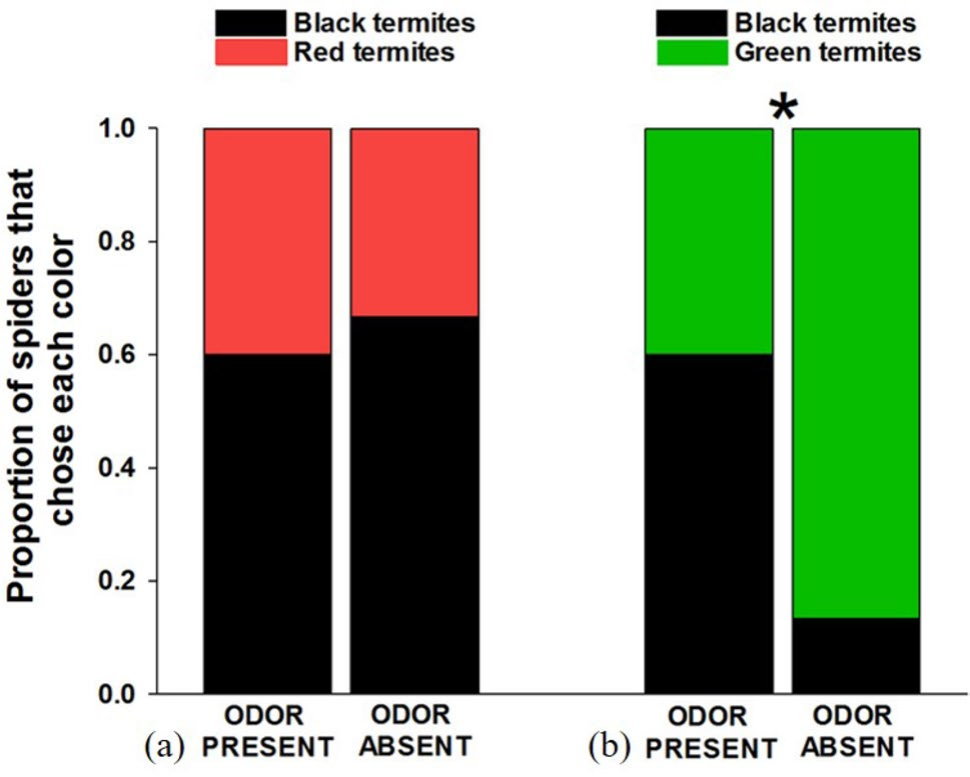
Results of choice tests between (**a**) red- and black-painted termites, and (**b**) green- and black-painted termites in the presence or absence of a defensive odor from the Asian ladybird beetle (*Harmonia axyridis*).

The presence or absence of the beetle odor had no effect on color preferences between red and black termites (n = 30; 6 female, 14 male, 10 juvenile; *X*^2^ = 0.144, *p* = 0.704; Fig. 4a). There was no evidence of color biases when the odor was either absent (*X*^2^ = 1.700, *p* = 0.192) nor present (*X*^2^ = 0.604, *p* = 0.437).

When the odor was present, the spiders were less likely to direct their first orientations towards red compared to when the odor was absent (*X*^2^ = 5.170, *p* = 0.023). The presence of the odor did not affect the time that it took to orient to the first termite (*F*_1,29_ = 0.112, *p* = 0.740), the time to attack the first termite (*F*_1,29_ = 0.093, *p* = 0.763), nor the spiders’ time to evaluate prey before attacking (*F*_1,29_ = 0.392, *p* = 0.536).

Neither sex/stage (*X*^2^ = 2.375, *p* = 0.305) nor size (*X*^2^ = 0.021, *p* = 0.885) affected spider color preferences.

#### Green versus black tests

Unexpectedly, we found that spiders in the presence of the beetle odor were more likely to attack green termites when the odor was present compared to when it was absent (n = 30; 10 female, 8 male, 12 juvenile; *X*^2^ = 7.459, *p* = 0.016; Fig. 4b). When the odor was absent, there was no evidence of color biases (*X*^2^ = 0.604, *p* = 0.437), but when the odor was present, spiders were more likely to attack green prey (*X*^2^ = 9.014, *p* = 0.003).

The presence of the odor did not affect the color that the spider first oriented to (*X*^2^ = 1.208, *p* = 0.272), the time that it took to orient to the first termite (*F*_1,29_ = 0.435, *p* = 0.515), the time to attack the first termite (*F*_1,29_ = 0.930, *p* = 0.343), nor the spiders’ time to evaluate prey before attacking (*F*_1,29_ = 0.611, *p* = 0.441).

Neither the sex/stage nor the size of spiders had any effect on color preferences (sex/stage: *X*^2^ = 1.485, *p* = 0.686; size: *X*^2^ = 0.502, *p* = 0.478).

### Experiment 4: Color choice tests in the presence/absence of odor from eastern lubber grasshoppers

#### Red versus black tests

**Figure 5:**
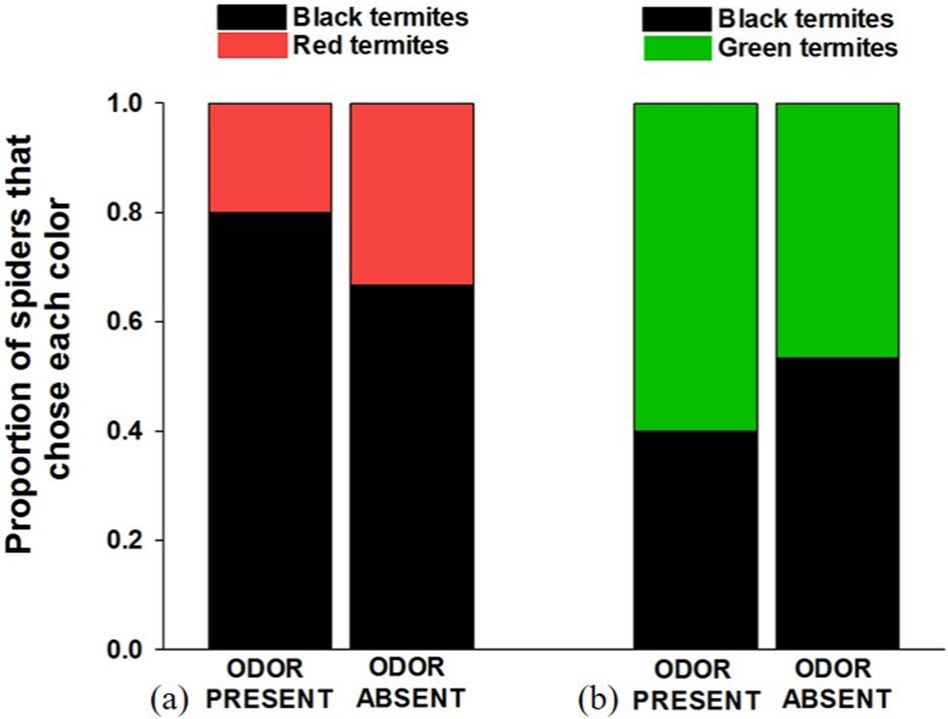
Results of choice tests between (**a**) red- and black-painted termites, and (**b**) green- and black-painted termites in the presence or absence of a defensive odor from the eastern lubber grasshopper (*Romalea microptera*).

The presence or absence of the grasshopper odor did not affect color preferences when given choices between red and black (n = 30; 7 = female, 14 = male, 9 = juvenile, *X*^2^ = 0.687, *p* = 0.407; Fig. 5a). There was no evidence of color biases when the odor was either absent (*X*^2^ = 1.700, *p* = 0.192) nor present (*X*^2^ = 5.782, *p* = 0.016).

The presence of the odor did not affect which color the spiders first oriented to (*X*^2^ = 0.000, *p* = 0.999), the time that it took to orient to the first termite (*F*_1,29_ = 0.003, *p* = 0.958), the time to attack the first termite (*F*_1,29_ = 0.024, *p* = 0.880), nor the spiders’ time to evaluate prey before attacking (*F*_1,29_ = 0.092, *p* = 0.764).

We did not detect effects of sex/stage (*X*^2^ = 2.204, *p* = 0.332) or size (*X*^2^ = 2.230, *p* = 0.122) on color preferences.

#### Green versus black tests

The presence or absence of the grasshopper odor did not affect color preferences when given choices between green and black (n = 30; 6 = female, 13 = male, 11 = juvenile; *X*^2^ = 0.537, *p* = 0.463; Fig. 5b). There was no evidence of color biases when the odor was either absent (*X*^2^ = 0.067, *p* = 0.796) nor present (*X*^2^ = 0.604, *p* = 0.437).

The presence of the odor did not affect the color that the spider first oriented to (*X*^2^ = 0.136, *p* = 0.712), the time that it took to orient to the first termite (*F*_1,29_ = 1.020, *p* = 0.321), the time to attack the first termite (*F*_1,29_ = 0.270, *p* = 0.607), nor the spiders’ time to evaluate prey before attacking (*F*_1,29_ = 0.070, *p* = 0.794).

Neither sex/stage nor size of spiders affected color preferences (sex/stage: *X*^2^ = 0.656, *p* = 0.720; size: *X*^2^ = 0.325, *p* = 0.568).

## Discussion

Our findings here show that the presence of defensive odors from multiple hemipteran insects (here, coreid bugs and stinkbugs) made *Habronattus* jumping spiders more likely to avoid red prey (compared to when the odor was absent). This study replicates and expands upon our previous work with *Habronattus*^18^ and is also consistent with the large body of avian work that focuses on the synergistic interactions between odor and color in aposematic prey^8–14,49^. Taken together, this suggests that birds and *Habronattus* jumping spiders may place remarkably similar selection pressures on the multimodal warning signals of their insect prey. For predators foraging in the field, the presence of aversive odors may provide a context for predators to interpret prey colors, allowing them to better avoid aposematic prey (e.g., see context-setting hypotheses^1^). However, this pattern did not hold up for all odors tested; when presented with defensive odors from either eastern lubber grasshoppers or Asian ladybird beetles, spiders did not reduce their attacks on the color red. In addition to red, we ran experiments to test if the same defensive odors affected preferences for the color green, a color not typically associated with aposematic prey (and therefore, not expected to be affected). As with our prior study^18^, we found that the presence of the odors had no effect on spider color preferences for the color green when using both hemipteran and orthopteran odors (yet, unexpectedly, in the presence of the Asian ladybird beetle odor, we found that spiders were more likely to attack green compared to black painted termites). Our results suggest that synergistic interactions between aversive odors and aposematic colors may be a general feature of *Habronattus* foraging ecology, at least when they encounter hemipteran prey, but may not apply to all colorful and chemically-defended prey.

Our finding that only hemipteran odors, and not the beetle and grasshopper odors, affected responses to the color red was contrary to our expectations because we had no a priori reason to expect only hemipteran odors to elicit this effect. However, odors produced by insects are diverse and are therefore unlikely to be equally effective or identical in their influence on predators. One possible explanation for our findings, while speculative, is that while the ladybird beetle and lubber grasshopper odors smelled aversive to us and have been hypothesized to act as deterrents to some predators (Asian ladybird beetles^40^, eastern lubber grasshoppers^36,50^), they may not be sufficiently aversive to the spiders to elicit an effect on color preferences (compared to the hemipteran odors). There is currently little data on the responses of multiple predators to these specific odors. However, in one study, hemolymph (the product of reflex-bleeding) from the Asian ladybird beetle was painted on the eggs of another ladybird beetle and this reduced egg cannibalism, suggesting that either the odor or taste of the hemolymph acts as a deterrent^40^. In another study, the odor from reflex blood of another ladybird beetle caused only a slight (and marginally significant) reduction in attacks by Japanese quail^51^. In studies of the defensive chemicals produced by eastern lubber grasshoppers, predatory ants were deterred by the odor of the secretions^36^ but predatory lizards were not^50^. Clearly more work is needed to understand the basic behavioral responses of suites of predators to the odors of various types of chemically-defended prey. This may allow us to better target which chemicals have the most aversive odors (from the predator’s perspective) and therefore may be most likely to interact with color preferences or responses to other aspects of complex warning displays.

Our study demonstrates that *H. trimaculatus* jumping spiders will reduce their attacks on red prey in the presence of certain defensive hemipteran odors, but more work needs to be done to tease apart the intricacies of how odor and color are interacting. In our previous study^18^, we found no evidence that the presence of the odor affects which color was detected first. However, in the present study, we found that in the presence of the two hemipteran odors, spiders were somewhat less likely (either significantly or nearly so) to direct their first orientations towards red prey (compared to black). This raises the possibility that lower attack rates on the red prey in the presence of the odor was a by-product of the spiders being less attentive to this color (rather than the defensive odor making the spiders *more* attentive to the color red, as theory surrounding multimodal warning displays would predict^1^). This possibility should be considered in the context of the unique visual system of jumping spiders that involve multiple pairs of functionally different eyes^26^. The orientation response in jumping spiders is mediated by a different set of eyes (the secondary or lateral eyes)^52^ which only have a single class of (green) photoreceptor cells^53^, making it unlikely that orientation responses would be influenced by chromatic cues. However, it is possible that responses mediated by these secondary eyes could be due to achromatic cues alone. Because these eyes have only green photoreceptors^53^, we would expect green-painted termites to be brighter and contrast less with our white experimental background compared with red- or black-painted termites. How odors might interact with both chromatic and achromatic cues detected by different sets of eyes will be an interesting line of future work. In assessing how color affects visual attention in the principal eyes, it may be more informative in future work to assess the total time spent orienting to the different colors rather than assessing which color first captures the spider’s attention. Also warranting further investigation is the unexpected finding that spiders were more likely to attack green compared to black painted termites in the presence of the Asian ladybird beetle odor (Experiment 3); we had no a priori reason to expect this odor to shift color biases for the color green, nor have we found anything in the literature that enables us to speculate on a functional explanation.

One question that remains from our study is how relevant these findings are to the predatory behavior of other species of jumping spiders. Might other jumping spiders similarly alter their color biases in the presence of hemipteran odors, or is this phenomenon specific to *H. trimaculatus*? Recent research has revealed that jumping spiders vary in their visual capacities, with different numbers and tunings of photoreceptor classes, that likely affects how they see and discriminate long wavelength colors (i.e., reds, oranges, yellows)^54^. Most jumping spiders are thought to have only limited color vision, with just UV and green photoreceptors; however, spiders in the genus *Habronattus* are an exception because they possess a retinal filter that shifts a subset of their photoreceptors to red, likely increasing their ability to see and discriminate long-wavelength colors like red that are typically associated with aposematic prey^27^. Ongoing research on salticid color vision suggests that some other species may have independently evolved alternative mechanisms for seeing and discriminating such long wavelength colors^54^. This raises the possibility that some select groups other than *Habronattus* might also have similar responses to odors and long-wavelength colors, such as red, in their prey. However, species without these mechanisms for long-wavelength color discrimination will likely not show the same odor-triggered aversion to red. Replication of our study across the salticid phylogeny, particularly in other key groups that can see and discriminate red, will help us understand how prey odor and color interact within this diverse and speciose group of predators.

The idea that an ecologically relevant odor might prime jumping spiders to pay attention to a particularly salient visual cue has been demonstrated in contexts other than aposematism. For example, the mosquito specialist, *Evarcha culicivora*, was better able to find cryptic mosquito lures in an experiment when they were first primed with mosquito odor^55^. And *Lyssomanes viridis* males primed with female pheromones were quicker to recognize images of conspecific females^56^. These examples highlight how the synergistic effects of odor and color likely vary by species and context. The general patterns of odor-triggered color biases that we detected in the present study were in response to lab-created combinations of defensive odors and colorful termites. Future experiments should extend his work to various naturally-occurring aposematic odor-color combinations informed by real insect warning displays in nature to explore the subtleties of how, when, and where odors have the strongest effect on responses to color.

Beyond salticids, more work still needs to be done to understand the role that other non-avian predators, specifically other terrestrial invertebrate predators, play in the evolution of aposematic colors and complex warning displays^6,55,56^. The diversity of invertebrate predators that feed on insects in nature is immense^16^, and yet they have largely been ignored despite the growing appreciation for their complex and varied cognitive abilities^57^. There is evidence that odor and color interact in other taxa for different contexts (e.g., pollination: bees and flowers^58^; butterflies and flowers^59^) and so this might be something that is more common across a range of predators than currently realized.

Within the field of animal behavior, there have been recent calls for more replication to help us assess the validity and generality of previous work^32,60,61^. This includes both exact replication (where an entire study is repeated with identical methods to the original) as well as partial, conceptual, and quasireplication, where studies are repeated with similar methods to help assess the generality of our conclusions^32,60^. Here we conducted a quasireplication of our previous work^18^; using identical experimental procedures, we assessed whether *H. trimaculatus*’ responses to odor and color were replicable when using different types of defensive odors, from different chemically defended insect groups. Our findings from the present study suggest that the odor-triggered color biases reported previously in *H. trimaculatus*^15^ are likely a general feature of their foraging ecology, as the results were nearly identical when experiments were repeated using three different hemipteran odors. Importantly, our study also allows us to identify the limits of this generality, as the expected patterns disappeared when using non-hemipteran odors. We can now extend this work further to ask what it is about hemipteran odors that trigger such color biases. Is it all hemipteran odors, or just those with a particular chemical makeup, or those that are delivered in a particular way? Such studies that replicate experiments across different contexts can help us improve our understanding of the subtleties of animal interactions.

## Supplementary information

Supplementary Figures.

## Data Availability

The datasets generated during and analyzed in this study are available in the Dryad repository (10.5061/dryad.8gtht76nk).

## References

[1] Rowe C, Halpin C (2013). Why are warning displays multimodal?. Behav. Ecol. Sociobiol..

[2] Endler J (1978). A predator’s view of animal colour patterns. Evol. Biol..

[3] Guilford T, Dawkins M (1991). Receiver psychology and the evolution of animal signals. Anim. Behav..

[4] Guilford, T. Predator psychology and the evolution of prey coloration. In: Crawley, M. Natural enemies: the population biology of predators, parasites and diseases. p. 375–394 (Oxford: Blackwell Scientific Publications, 1992).

[5] Rowe, C. Receiver psychology and the evolution of multicomponent signals. *Anim. Behav*. **58**, 921–931. 10.1006/anbe.1999.1242 10564594

[6] Miller C, Bee M (2012). Receiver psychology turns 20: is it time for a broader approach?. Anim. Behav..

[7] Rowe C (2013). Receiver psychology: a receiver’s perspective. Anim. Behav..

[8] Marples N, Roper T (1996). Effects of novel colour and smell on the response of naive chicks towards food and water. Anim. Behav..

[9] Rowe C, Guilford T (1996). Hidden colour aversions in domestic chicks triggered by pyrazine odours of insect warning displays. Nature.

[10] Rowe C, Guilford T (1999). The evolution of multimodal warning displays. Evol. Ecol..

[11] Rowe C, Guilford T (1999). Novelty effects in multimodal warning signals. Anim. Behav..

[12] Jetz W, Rowe C, Guilford T (2001). Non-warning odors trigger innate color aversions—as long as they are novel. Behav. Ecol..

[13] Lindström, L., Rowe, C. & Guilford, T. Pyrazine odour makes visually conspicuous prey aversive. *Proc. R. Soc. London., Ser. B* **268**, 159–162 (2001). 10.1098/rspb.2000.1344 PMC1088585 11209885

[14] Kelly D, Marples N (2004). The effects of novel odour and colour cues on food acceptance by the zebra finch *Taeniopygia guttata*. Anim. Behav..

[15] Siddall E, Marples N (2008). Better to be bimodal: the interaction of color and odor on learning and memory. Behav. Ecol..

[16] Symondson W, Sunderland K, Greenstone M (2002). Can generalist predators be effective biocontrol agents?. Annu. Rev. Entomol..

[17] Raška J, Štys P, Exnerová A (2018). Perception of olfactory aposematic signals by jumping spiders. Ethology.

[18] Vickers M, Taylor L (2018). Odor alters color preference in a foraging jumping spider. Behav. Ecol..

[19] Taylor L (2014). Colour use by tiny predators: jumping spiders show colour biases during foraging. Anim. Behav..

[20] Taylor L (2016). Flexible color learning in an invertebrate predator: *Habronattus* jumping spiders can learn to prefer or avoid red during foraging. Behav. Ecol..

[21] Kauppinen J, Mappes J (2003). Why are wasps so intimidating: field experiments on hunting dragonflies (Odonata: *Aeshna grandis*). Anim. Behav..

[22] Bowdish T, Bultman T (1993). Visual cues used by mantids in learning aversion to aposematically colored prey. Am. Nat..

[23] Ramesh A (2016). Similar yet different: differential response of a praying mantis to ant-mimicking spiders. Biol. J. Linn. Soc..

[24] Powell E (2019). Prey colour biases in jumping spiders (Habronattus brunneus) differ across populations. Ethology.

[25] Jackson R, Pollard S (1996). Predatory behavior of jumping spiders. Annu. Rev. Entomol..

[26] Harland D, Jackson R (2000). 'Eight-legged cats' and how they see: a review of recent research on jumping spiders (Araneae: Salticidae). Cimbebasia.

[27] Zurek D (2015). Spectral filtering enables trichromatic vision in colorful jumping spiders. Curr. Bio..

[28] Griswold, C. A revision of the jumping spider genus *Habronattus* FOP-Cambridge (Araneae, Salticidae), with phenetic and cladistic analyses. (Univ of California Press 1987).

[29] Aldrich R (1988). Chemical ecology of the Heteroptera. Annu. Rev. Entomol..

[30] Dettner K (1987). Chemosystematics and evolution of beetle chemical defenses. Annu. Rev. Entomol..

[31] Stevens M, Ruxton G (2012). Linking the evolution and form of warning coloration in nature. Proc. Royal Soc. B..

[32] Nakagawa S, Parker T (2015). Replicating research in ecology and evolution: feasibility, incentives, and the cost-benefit conundrum. BMC Biol..

[33] Sloggett J (2011). The chemical ecology of *Harmonia axyridis*. Biocontrol.

[34] Schowalter T (2018). Biology and management of the eastern lubber grasshopper (Orthoptera: Acrididae). J. Integr. Pest. Manag..

[35] Pasteels M, Gregoire J-C (1983). The chemical ecology of defense in arthropods. Ann. Rev. Entomol..

[36] Jones C (1989). Reduction in diet breadth results in sequestration of plant chemicals and increases efficacy of chemical defense in a generalist grasshopper. J. Chem. Ecol..

[37] Blum M (1992). Ingested allelochemicals in insect wonderland: a menu of remarkable functions. Am. Entomol..

[38] Hough-Goldstein J, Cox J, Armstrong A (1996). *Podisus maculiventris* (Hemiptera: Pentatomidae) predation on ladybird beetles (Coleoptera: Coccinellidae). Fla. Entomol..

[39] Glendinning J (2007). How do predators cope with chemically defended foods?. Bio. Bull-US..

[40] Sato S, Kushibuchi K, Yasuda H (2009). Effect of reflex bleeding of a predatory ladybird beetle, *Harmonia axyridis* (Pallas) (Coleoptera: Coccinellidae), as a means of avoiding intraguild predation and its cost. Appl. Entomol. Zool..

[41] Millar, J. Pheromones of true bugs. In: The chemistry of pheromones and other semiochemicals II. p. 37–84 (Berlin: Springer, 2005).

[42] Capinera, J. & Scherer C. Featured Creatures: *Romalea microptera* (Palisot de Beauvois) (Insecta: Orthoptera: Romaleidae). University of Florida Institute of Food and Agricultural Sciences. EDIS publication: **EENY-6** (2016).

[43] Hill D (2006). Learned avoidance of the large milkweed bug (Hemiptera, Lygaeidae, *Oncopeltus fasciatus*) by jumping spiders (Araneae, Salticidae, *Phidippus*). Peckhamia..

[44] Barão K, Ferrari A, Adami C, Grazia J (2017). Diversity of the external thoracic scent efferent system of Carpocorini (Heteroptera: Pentatomidae) with character selection for phylogenetic inference. Zool. Anz..

[45] Aldrich R, Yonke T (1975). Natural products of abdominal and metathoracic scent glands of coreoid bugs. Ann. Entomol. Soc. Am..

[46] Aldrich R, Blum M, Lloyd H (1978). Pentatomid natural products. J. Chem. Ecol..

[47] Whitman D, Jones C, Blum S (1992). Defense secretions production in lubber grasshoppers (Orthoptera: Romaleidae): influence of age, sex, diet, and discharge frequency. Ann. Entomol. Soc. Am..

[48] Taylor L, McGraw K (2013). Male ornamental coloration improves courtship success in a jumping spider, but only in the sun. Behav. Ecol..

[49] Skelhorn J, Rowe C (2006). Avian predators taste–reject aposematic prey on the basis of their chemical defence. Biol. Lett..

[50] Hatle J, Townsend V (1996). Defensive secretion of a flightless grasshopper: failure to prevent lizard attack. Chemoecology.

[51] Marples N, van Veelen W, Brakefield P (1994). The relative importance of colour, taste and smell in the protection of an aposematic insect *Coccinella septempunctata*. Anim. Behav..

[52] Land M (1971). Orientation by jumping spiders in the absence of visual feedback. J. Exp. Biol..

[53] Yamashita S, Tateda H (1976). Spectral sensitivities of jumping spider eyes. J. Comp. Physiol. A..

[54] Outomuro D (2019). The evolution of colour vision across jumping spiders. Integr. Comp. Biol..

[55] Cross F, Jackson R (2009). Cross-modality priming of visual and olfactory selective attention by a spider that feeds indirectly on vertebrate blood. J. Exp. Biol..

[56] Ruxton G, Sherratt T, Speed M (2004). Avoiding attack: the evolutionary ecology of crypsis, warning signals and mimicry.

[57] Giurfa M (2013). Cognition with few neurons: higher-order learning in insects. Trends Neurosci..

[58] Leonard A, Masek P (2014). Multisensory integration of colors and scents: insights from bees and flowers. J. Comp. Physiol. A..

[59] Yoshida M (2015). Plant scents modify innate colour preference in foraging swallowtail butterflies. Biol. Lett..

[60] Kelly C (2006). Replicating empirical research in behavioral ecology: how and why it should be done but rarely ever is. Q. Rev. Biol..

[61] Ihle M, Winney I, Krystalli A, Croucher M (2017). Striving for transparent and credible research: practical guidelines for behavioral ecologists. Behav. Ecol..

