# Supplementary material for: Hemipteran defensive odors trigger predictable color biases in jumping spider predators
Source: Sci Rep. 2020 Dec 14;10:21898. doi: 10.1038/s41598-020-78952-5 (PMC7736339; doi:10.1038/s41598-020-78952-5)
Supplement: Supplementary file 1 — Supplementary Figures. [file 41598_2020_78952_MOESM1_ESM.pdf]

Supplementary material

Vickers, ME and Taylor, LA

Hemipteran defensive odors trigger predictable color biases in jumping spider predators

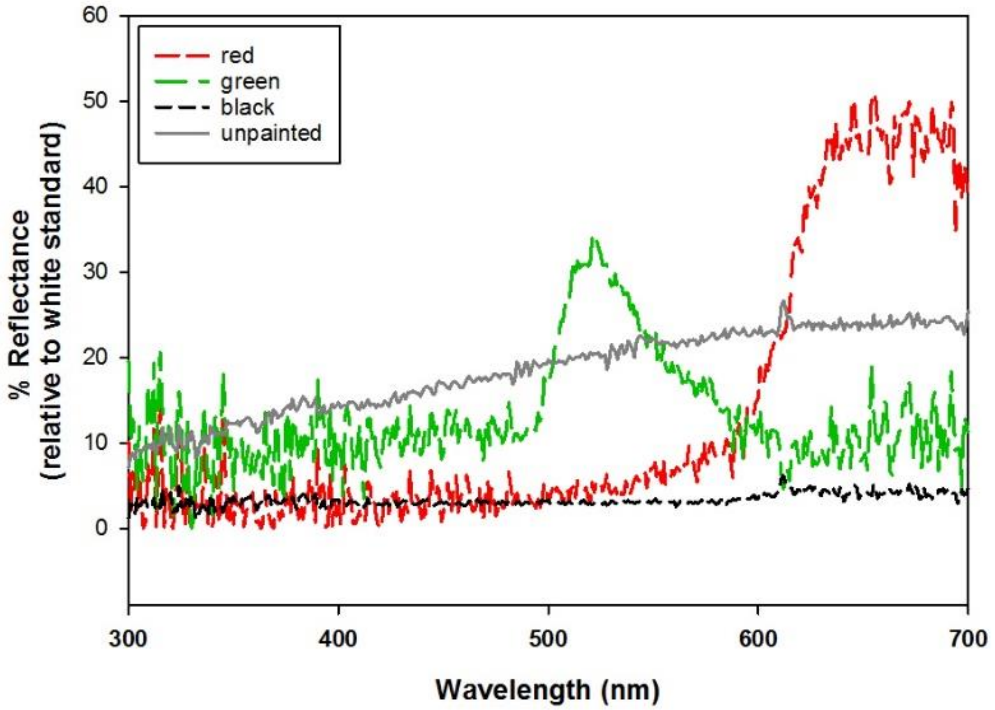

Figure S1. Spectral properties of artificially-painted (red, green, and black) termites used in color choice tests. Unpainted (naturally-colored) termites are shown for comparison. Spectral curves show the mean values for 10 individuals of each color. Spectral properties were measured using a UV-vis spectrophotometer (USB 2000 with PX-2 pulsed xenon light source, Ocean Optics, Dunedin, FL) with the measurement probe held perpendicular to the colored surface and a measurement pin attached to the probe to ensure a consistent distance between the probe and sample. Measurements were taken relative to a Spectralon diffuse reflectance white standard (Labsphere Inc., North Sutton, NH) (reproduced from Vickers and Taylor 2018).

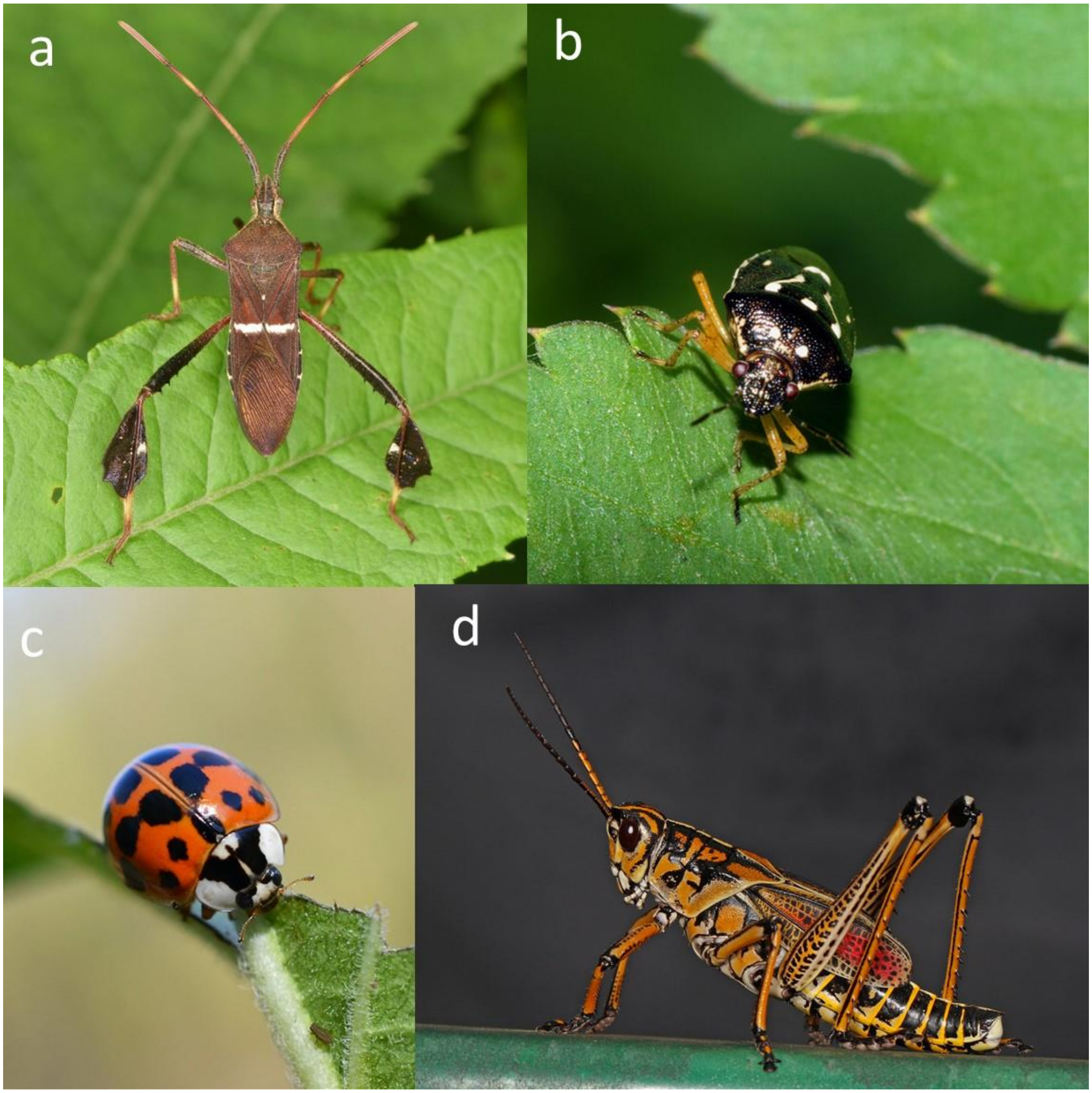

Figure S2. Insect species used to collect defensive odors that were used during color choice tests. (a) *Leptoglossus phyllopus* (eastern leaf-footed bug, Hemiptera: Coreidae), (b) *Mormidea pama* (grass stinkbug, Hemiptera: Pentatomidae), (c) *Harmonia axyridis* (Asian ladybeetle, Coleoptera: Coccinellidae), (d) *Romalea microptera* (eastern lubber grasshopper, Orthoptera: Acrididae). Images obtained from creativecommons.org; detailed credits provided below.

Image credits for Fig. S2.

(a) "Eastern Leaf-footed Bug- Leptoglossus phyllopus" by Wedontneedfeatherstofly is licensed with CC BY 2.0. To view a copy of this license, visit https://creativecommons.org/licenses/by/2.0/

(b) "Mormidea pama" by Larah McElroy is licensed with CC BY-NC 2.0. To view a copy of this license, visit https://creativecommons.org/licenses/by-nc/2.0/

(c) "Multicolored Asian Ladybird Beetle (Harmonia axyridis)" by acryptozoo is licensed with CC BY 2.0. To view a copy of this license, visit https://creativecommons.org/licenses/by/2.0/

(d) "Eastern Lubber Grasshopper - Romalea microptera, Mississippi Sandhill Crane National Wildlife Refuge, Gautier, Mississippi" by Judy Gallagher is licensed with CC BY 2.0. To view a copy of this license, visit https://creativecommons.org/licenses/by/2.0/
